# Efficacy of TNF blockers from the perspective of growth velocity: slovak experience

**DOI:** 10.1186/1546-0096-12-S1-P148

**Published:** 2014-09-17

**Authors:** Pavol Mrážik, Elena Košková, Veronika Vargová

**Affiliations:** 1 Department of Paediatrics and Adolescent Medicine, Šafárik University and Children Faculty Hospital in Košice, Košice, Slovakia; 2 Department of Pediatric Rheumatology, National Institute of Rheumatic Diseases, Piešťany, Slovakia

## Introduction

In patients with polyarticular course of juvenile idiopathic arthritis (JIA) both, ongoing chronic inflammation with high levels of pro-inflammatory cytokines and corticosteroid treatment, may affect linear growth adversely, potentially leading to irreversible growth retardation. Biologic treatment of JIA may have the potential to enable normal growth in children with JIA.

## Objectives

To retrospectively evaluate clinical efficacy, from perspective of growth velocity as a sensitive marker of well-being in children, of biologics in children with JIA in both centers in Slovakia.

## Methods

34 patients (17 girls, 17 boys) with DMARDs-resistant polyarticular JIA receiving TNFα blocker (28 etanercept, 6 adalimumab) + methotrexate were evaluated during their first year of biologic therapy. The mean age at anti-TNF treatment initiation was 10.52 (range 2.98-17.77). Median duration of disease was 4.42 years (range 0.89-10.63), 24 patients received corticosteroids. Control group, consisted of 21 patients (12 boys, 9 girls) with polyarticular course of JIA non-responsive to NSAIDs therapy and intraarticular corticosteroid injections, was evaluated during their first year of DMARDs treatment. Same inclusion and exclusion criteria, e.g. age and Tanner staging, were applied for both groups. Growth velocity was defined by change of height in standard deviation score (SDS). A positive value >0.1 indicated catch-up and negative value <-0.1 indicated impaired growth. Values in the range -0.1 to 0.1 were labeled as "steady growth".

## Results

Efficacy of combination TNFα blocker + MTX treatment evaluated by ACR Pediatric 30 is increasing during the first year. In time of year control 34/31/25 patients reach criteria at least for ACR Pediatric 30/50/70 respectively. The number of joints with acute arthritis decreased by 91.9 % (from 309 to 25) compared to 91.33 % (from 173 to 15) in control group. TNFα blocker + MTX combination also shows a rapid corticosteroid-sparing effect. Only 4 patients received corticosteroids at the start of DMARDs treatment in control group. Clinical response in TNFα blocker + MTX group was accompanied by catch-up in 18 patients, "steady growth" and impaired growth were observed in 8 cases both. Clinical response in control group was accompanied by catch-up only in 3 cases, "steady growth" and impaired growth were observed in 6 and 8 patients respectively. The difference in growth velocity was statistically significant (0.106 ± SD 0.439 vs. -0.139 ± SD 0.385, t = 2.101 df = 53, p = 0.040).

## Conclusion

Combination of TNFα blocker + methotrexate is highly effective in polyarticular course of DMARDs-resistant JIA. The clinical response is accompanied by an increase in growth velocity (catch-up) in most cases. The growth velocity compared with control group was on the border of statistical significance.

## Disclosure of interest

None declared.

